# *Histoplasma capsulatum* causing sinusitis: a case report in French Guiana and review of the literature

**DOI:** 10.1186/s12879-018-3499-5

**Published:** 2018-11-26

**Authors:** C. Nabet, C. Belzunce, D. Blanchet, P. Abboud, F. Djossou, B. Carme, C. Aznar, M. Demar

**Affiliations:** 1 AcademicLaboratory of Parasitology and Mycology, Andrée Rosemon Hospital, 97306 Cayenne, French Guiana; 2 Unit of infectious diseases and Tropical medicine, Andrée Rosemon Hospital, Cayenne, French Guiana; 3 Faculty of Medicine, University of French Guiana, EA 3593 EPaT, Amazonian Ecosystems and Tropical Disease, Cayenne, French Guiana

**Keywords:** *Histoplasma capsulatum*, AIDS, Sinusitis, Sinus fungus ball, Review

## Abstract

**Background:**

American histoplasmosis is a mycosis caused by *Histoplasma capsulatum*. A variety of clinical features of histoplasmosis have been commonly described ranging from asymptomatic infections to severe pulmonary infections. In immunocompromised individuals, progressive disseminated forms are frequent, leading to fatal outcome if untreated. However, *H. capsulatum* sinusitis is unusual with a few cases documented over the last three decades and may be underdiagnosed or confused with other fungal aetiologies, especially outside endemic regions.

**Case presentation:**

In this study, we report an atypical case of *Histoplasma capsulatum* sinus fungus ball-like in a patient with Acquired Immunodeficiency Syndrome due to Human Immunodeficiency Virus complicated by a disseminated histoplasmosis with a death ending. Diagnosis relied on CT-Scan imaging and on both direct examination of *H. capsulatum* yeast forms (Gomory methenamine Grocott) in the sinus specimen (aspirate, biopsy) and on positivity of the culture further confirmed by qPCR.

**Conclusions:**

Since last few decades, among the eight reviewed patients, *H. capsulatum* sinusitis occurred mostly in HIV-immunocompromised patients and for three cases as a sinusitis with disseminated histoplasmosis. Even if this is a rare clinical presentation, its diagnosis is crucial as it could represent an early expression of an *Histoplasma capsulatum* exposure that can evolve into a disseminated fatal infection when immunity decreases.

## Background

Sinusitis, also called rhinosinusitis and referred as symptomatic inflammation of the nasal and paranasal sinus mucosa, is a very common affection in general population. The most frequently reported pathogens are bacteria and viruses [1, 2]. The recent well-defined entity named fungal rhinosinusitis whilst less prevalent is emerging, especially in tropical areas [3, 4]. Diverse clinical presentations and histopathology features of fungal rhinosinusitis are described based on the mode of fungal invasion of the tissues, probably depending on the variety of host interactions [5–8]. The non-invasive form consists on fungall ball and allergic fungal rhinosinusitis whereas the invasive form includes acute, chronic, and chronic granulomatous rhinosinusitis. Fungal rhinosinusitis is commonly due to *Aspergillus spp* but other fungus species such as yeasts (*Cryptococcus spp.*, *Candida spp*.), molds (Dematiaceous *species, Rhizopus spp*., *Fusarium spp* and *Scedosporium spp*.) have been reported mainly in HIV-AIDS patients [5, 6, 9, 10]. Few reports involving *Histoplasma capsulatum (HC),* an environmental and dimorphic fungus responsible for granulomatous disease are signed in the literature. This opportunist pathogen is isolated from endemic areas such as North, Central and South America, Africa, Asia and Australia with two pathogenic varieties in human, the worldwide *H. capsulatum var. capsulatum* and *H. capsulatum var. duboisii* isolated only in Africa [11]*.*

The severity of the disease mainly depends on the inoculum size and the immunosuppression level allowing dissemination of the infection from lungs to lymph nodes or visceral involvement through the bloodstream [11–15]. This can lead to progressive disseminated histoplasmosis that can be fatal when therapy is delayed and when misdiagnosed [11, 14]. Though, rapid and accurate biological diagnostic of histoplasmosis is challenging as the absence of specific symptoms can lead to possible confusion with other opportunistic microbial agents such as tuberculosis [16]. This is even more obvious in case of atypical localizations. In French Guiana, histoplasmosis is known as a major opportunistic infection and one of the main causes of AIDS-related death, along with tuberculosis [14, 16–18]. First case of Histoplasmosis sinusitis was reported in 1993 from California, USA [19]. Globally, since the last three decades only 8 cases have been documented. Herein, such a case is reported in an HIV related-immunosuppressed adult and we then presented a comprehensive review of literature and analysed the previously reported cases.

## Case presentation

### Clinical presentation

A 50-year-old woman native of Haiti presented to the Cayenne hospital in French Guiana with complaints of persistent fever, mild cough, asthenia and anorexia. She reported medical consultation three months before for febrile nasal obstruction and cough treated by amoxicillin-clavulanic acid without ameliorations. She lived in French Guiana for 20 years.

**Fig. 1:**
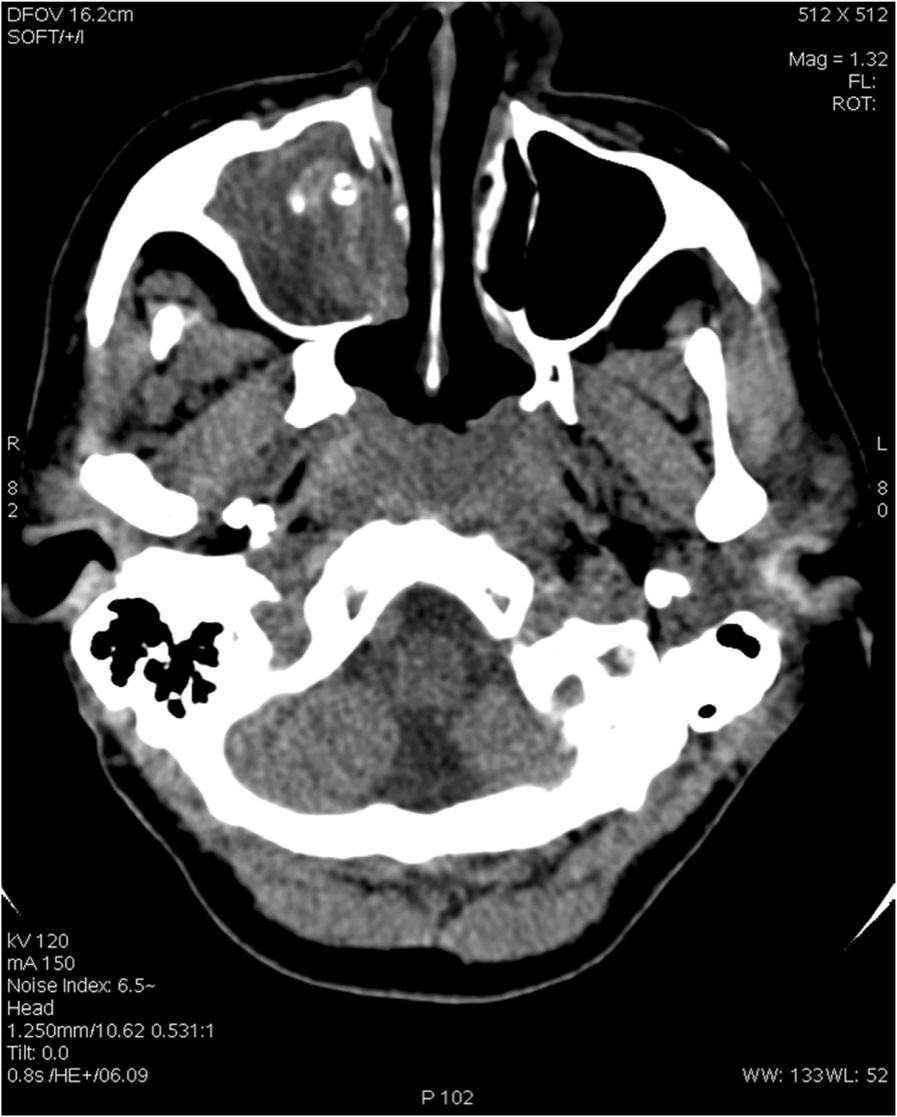
Representative Computerized-Tomography Scan (CT-Scan) of the sinuses showing complete opacification of right maxillary sinus with flocculent calcifications and an absence of bone erosion

Diagnosis of HIV infection was further achieved, showing a high viral replication level (HIV viral load = 7,300,000 copies/mL) along with a deeply immunocompromised status (CD4 count = 22 cells/mm3). A full-body computer tomography scanner (CT scan) showed a complete opacification of right maxillary sinus filled with flocculent calcifications (Fig. 1). This typical aspect along with an absence of osteolysis or osteocondensation was evocative of an *Aspergillus spp.* fungus ball. Additionally, CT scan showed bilateral inferior alveolar opacities (also visible during chest radiography), hepatosplenomegaly and disseminated lymph nodes up to 2 cms.

### Clinical and laboratory findings

Diagnosis protocol included for microbiological examinations: sputum, bronchoalveolar lavage (BAL), maxillary sinus puncture, bone marrow, osteomedullar, node and hepatitis biopsies. Potassium hydroxide (KOH) direct examination and May-Grünwald Giemsa (MGG) stained smears were negative except for the sputum and the BAL. They showed an association of yeast and a gram-negative bacterium further identified in culture as *Candida albicans, Klebsiella pneumonia* and *Pseudomonas fluorescens*. Histopathology examination including Gomory Methenamine-Silver Grocott (GG) and Periodic Acid Schiff (PAS) staining have been practiced on the liver, the osteomedullar biopsy and the BAL fluid and were negative. Serologic analysis revealed no detection of *Aspergillus spp.* galactomannan antigen nor specific antibodies for *Histoplasma* or *Leishmania.*

### Treatment and outcome of the patient

Patient suddenly developed a respiratory distress syndrome along with a severe sepsis-like infection, after a prolonged well-supported fever. Antifungal therapy with Voriconazole was introduced due to presumption of an *Aspergillus spp.* maxillary infection that may be associated with pulmonary aspergillosis. Antimicrobial therapy with Piperacillin-tazobactam was introduced to cover a potential bacterial pulmonary infection (*Klebsiella spp.* and *Pseudomonas spp*.). Finally, the patient rapidly died of a respiratory distress syndrome, three days after the beginning of the pharmacological treatment and before the *H. capsulatum* infection was diagnosed.

### Confirmed diagnosis

**Fig. 2:**
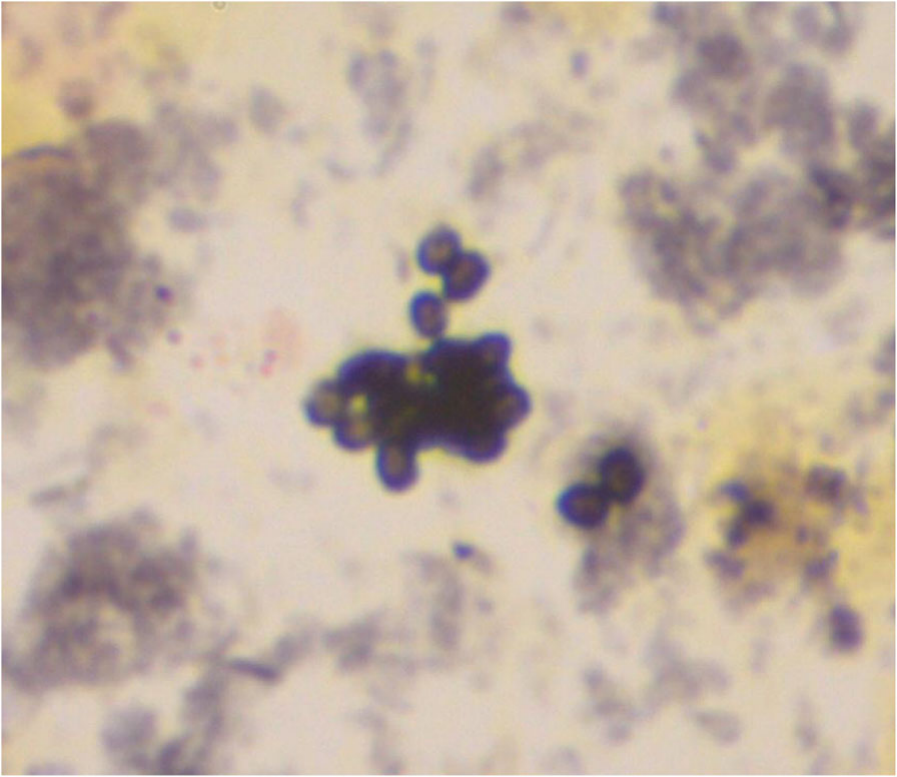
Gomory Methenamine-Silver Grocott staining of the sinus aspirate showing ovoid 2–4 μm yeasts compatible with *Histoplasma capsulatum var. capsulatum*

Indeed, *H. capsulatum* was isolated from the sinusitis aspirate and from cervical adenopathy biopsy after 20 and 15 days of culture, respectively using Sabouraud/chloramphenicol/gentamicin agar media at 30 °C. The other fungal cultures (bone marrow, liver biopsy, blood, urine, BAL and expectorations) remained sterile after 2 months of incubation. Retrospectively, after a slide discoloration, a GG staining was performed on sinus aspirate and allowed to identify ovoid 2–4 μm yeasts evocative of *Histoplasma capsulatum var. capsulatum,* previously not visible during MGG staining (Fig. 2). Additionally, a *H. capsulatum* real-time PCR analysis developed by the Academic Laboratory of Parasitology and Mycology, Cayenne Hospital, French Guiana [20] was retrospectively performed on expectorations, BAL fluid and node biopsy and tested positive, confirming the diagnosis.

Several underlying pathologies have been revealed during post-mortem analysis. First, lymphoma diagnostic was evocated following direct examination of bone marrow, osteomedullar and node biopsies and was confirmed as a B lymphocyte-depleted Hodgkin’s disease by immunophenotyping (CD20+ CD3+ CD30+ ki67+). Then, disseminated *Mycobacterium avium* infection was diagnosed by PCR on positive culturing of BAL, bone marrow, liver biopsy and node biopsy.

### Literature review

A search for published cases of *H. capsulatum* sinusitis was conducted in PubMed database using the keywords “*Histoplasma capsulatum*”, “sinusitis”, “rhinosinusitis”, “histoplasmosis”. Only cases in which imaging confirmed sinusitis and *H. capsulatum* was documented by histopathologic direct examination or culture were included in the review. Studies in any language were considered and reviewed. Extracted data included age and gender, patient origin, underlying disease, clinical signs, symptoms duration, site of infection, direct examination and culture results, CT scan images of the sinus, treatment and outcome.

Eight articles reporting 8 cases of *H. capsulatum* sinusitis from 1993 to the present were included. These cases are summarized in Table 1 [19, 21–27].

**Table 1:** Reported cases of sinusitis caused by *H. capsulatum*

Case number	Gender/age	Underlying disease	Patient origin	Clinical signs	Symptoms duration	Infection site	Direct examination by GG	Culture result (*tested sample*)	Sinus/oral lesions (CT-Scan)	Treatment	Outcome	Reference
							(*tested sample*)					
1	Male/41	HIV	USA	Palatal perforation (ulcerative and granulomatous lesions) Pansinusitis	–	Sinus, palate	Positive	Positive	–	Palatal obturator, AMB	Complete cure	19
2	Female/37	HIV	South Africa	Swelling of the upper lip, Painful mouth, Bleeding gums of palate and buccal mucosa, Cervical bilateral lymphadenopathies	9 months	Sinus, palate	Positive *(palatal biopsy)*	Positive *(palatal biopsy)*	Hard palate (destruction), Maxillary sinuses, right nasal cavity (opacification)	ITR	Improvement	21
3	Male/39	HIV	USA	Fever, Cough, Nasal congestion, Headache, Submandibular lymph node, Skin rash (Maculo-papular)	2 months	Sinus, skin	Positive *(sinus aspirate, skin biopsy)*	Positive *(sinus aspirate, skin biopsy)*	Maxillary sinuses (complete opacifiaction), fontal sinuses (partial opacification)	ITR	Improvement	22
4	Male/40	HIV	Brasil	Fever, Nasal congestion, Rhinorrhea, Hyperemia palpebral, cutaneous alteration	3 months	Sinus, skin	Positive *(sinus biopsy)*	Positive *(sinus aspirate, skin biopsy)*	Right maxillary and ethmoid sinuses (complete opacification), frontal sinuses (partial opacification)	ITR	Complete cure	23
5	Male/35	HIV	USA	Fever, Gingival pain, Frontal headache, Pneumopathy	8 months	Sinus, lungs	Positive *(sinus biopsy)*	Positive *(sinus aspirate, sinus biopsy)*	Maxillary and ethmoid sinuses (complete opacification)	POS	Complete cure	24
6	Male/76	CLL	Brasil	Facial pain, epistaxis, fever, facial erythema, swelling	2 months	Sinus	Positive *(sinus aspirate)*	Positive *(sinus aspirate)*	Right maxillary, frontral, ethmoïdal sinuses (partial opacification)	AMB	Improvement	25
7	Male/90	No	India	Throat bleeding, Nasal congestion, Rhinorrhea, Headache Palatal perforation	6 months	Sinus, Palate	Positive *(oral biospsy)*	Positive *(oral biopsy)*	Hard palate (destruction), right maxillary sinus (opacification)	ITR	Improvement	26
8	Male/39	HIV, tuberculosis	Morocco	Fever, Cough, Nasal congestion, Rhinorrhea, Epistaxis, Headhache, Skin rash (papular and nodular), Hepatosplenomegaly	3 months	Sinus, skin	Positive *(sinus aspirate, skin biopsy*)	Positive *(sinus aspirate, skin biopsy)*	Maxillary, ethmoid sinuses (complete opacification), frontal sinuses (partial opacification)	AMB	Complete cure	27

*CLL* Chronic Lymphocytic Leukaemia, *ITR* Itraconazole, *AMB* Amphotericine B, *POS* Posaconazole

## Discussions and conclusions

In this study, fungal rhinosinusitis related to *H. capsulatum* is very uncommon and remains anecdotal as only eight cases have been published between 1993 and 2016. In the present case, it involves *Histoplasma capsulatum var capsulatum* and clinically presents as an atypical fungus ball-like in a deeply immunosuppressed patient with several co-morbidities.

### Risk factors for developing *Histoplasma* fungal rhinosinusitis and clinico-epidemiological aspects

In our review, cases were reported from North America (*n* = 3), South America (*n* = 2), North Africa (*n* = 1), South Africa (n = 1) and India (n = 1). The mean age was 49 years, ranging from 35 to 90 years. Most cases of *H. capsulatum* sinusitis including the present case were described mostly in men (sex ratio H/F: 7/1) and immunocompromised host except for one of them (case 7). Indeed, six patients had HIV infection (cases 1, 2, 3, 4, 5, 8) and one a Chronic Lymphocytic Leukaemia (case 6). These features are in contrast with reported cases of typical sinus fungus ball. They usually occur in immunocompetent individuals and are more prevalent in women who had a prior history of dental surgery (iatrogenic oro-antral communication secondary to dental extraction, periodontal destruction or most often endodontic treatment with filling of the dental canal) [8–10].

According to the published sinus fungus ball findings, maxillary sinus is the most frequently involved sinus and multiple sinus locations are uncommon. In our review, other sinusal locations such as ethmoid and frontal were associated with the maxillary sinus for 5 cases (3, 4, 5, 6, 8). Moreover, multiples sites of infection included the association of sinus/palate (cases 1, 2, 7), sinus/lung (case 5), sinus/skin (cases 3, 4, 8), sinus/cervical adenopathy/lung (present case). Only one case reported an unique sinusal location (case 6). Symptoms duration varied from 2 to 9 months (mean of 5 months) and main symptoms were fever (*n* = 5), headache (*n* = 4), nasal congestion (*n* = 4), rhinorrhoea (*n* = 3), palatal ulceration (*n* = 3), oral bleeding (*n* = 2), skin rash (n = 3) and pneumopathy (*n* = 1).

### Pathogenesis of Histoplasma fungal rhinosinusitis

Fungal sinusitis is divided into invasive and non-invasive forms [8, 10]. Fungus ball of the paranasal sinuses is defined as the non-invasive accumulation of dense fungal concrements in sinusal cavities, most often the maxillary sinus. Clinicopathological criteria for diagnosis of fungus ball include, according to deShazo’s et al. [28]: 1) Radiological evidence of sinus opacification with or without associated flocculent calcifications, 2) Mucopurulent, cheesy or clay-like material within a sinus, 3) a matted, dense conglomeration of hyphae separate from but adjacent to sinus respiratory mucosa 4) A chronic inflammatory response in the mucosa adjacent to fungal elements and 5) no histological evidence of fungal invasion of mucosa on GG.

Unlike invasive rhinosinusitis, sinus fungus ball never causes progression or invasion [8, 10]. However, to explain the multiple sites of infection in 7 of the 8 reported cases (1,5,7,8) and the present case, we could mention two different mechanisms. First, for non-contiguous sites, the dissemination may have resulted of a primary exposure to *H. capsulatum* by direct inhalation of spores. Probably, an acute pulmonary disease could have happened at exposure time and may have been initially recovered by immunity (granulomatous lesion). This was supposed for cases 3–5, 8 and present case that displayed a several months history of nasal obstruction, rhinorrhoea, headache and cough. A secondary progressive disseminated fungal infection was highly suspected for cases 3–5, 8 and present case, as patients presented fever, chest radiograph abnormalities (3, 5, 8, present case, not reported in Table 1), hepatosplenomegaly (8, present case), skin lesions (3, 4, 8) and disseminated lymph nodes (8, present case). It was confirmed for cases 3, 4, 8 and present case as *H. capsulatum* was found in culture from both sinus specimen and a specimen from a distant site (skin biopsy, cervical lymph node). In present case, *H. capsulatum* was retrospectively revealed on respiratory specimens with real-time PCR analysis; This pulmonary localisation may have been the potential source of fungus dissemination to cervical lymph node through the hematogenous route. The other mechanism for the development of the sinusitis may be a local extension from a contiguous source [7, 10]. Indeed, for cases 1, 2 and 7, a prior palatal fungal ulcer may have eroded the hard palate and lead to secondary sinus infection by direct extension. When reviewing *H. capsulatum* sinusitis cases, no cases reporting with an odontogenic pathway mechanism have been described whereas it is a principal risk factor described for typical sinus fungus ball [8, 10].

### Challenge in the diagnosis and utility of new diagnostic tools

Because histoplasmosis can have an extended incubation period and may goes undiagnosed for a long time before the worsening of symptoms, its diagnosis remains a real challenge. Moreover, the clinical presentation is misleading because usually non-specific and sometimes uncommon as reported in our review.

The present case emphasises the difficulties in diagnosing *H. capsulatum* from the direct examination. It highlights the better performance of GG staining for some samples, as we missed the diagnosis by using the MGG staining. In the review, direct microscopic examination of sinus aspirate (cases 3,6,8) staining by GG easily revealed *Histoplasma capsulatum* yeasts. Additionally, *H. capsulatum* sinusitis may be confused with an *Aspergillus spp.* fungus ball. Indeed, our patient’s radiological findings were strongly suggestive for an *Aspergillus spp.* sinus fungus ball showing complete opacification of maxillary sinus associated with flocculent calcifications. Another *Aspergillus spp.* fungus ball confusing element was the mucopurulent, clay-like aspect of the material within the sinus. Thus, in case of suggestive aspect of *Aspergillus spp.* fungus ball, it seems relevant to search for atypical fungal pathogens such as *H. capsulatum*, especially in immunocompromised host and in endemic areas.

We could not detail the fungus ball clinical form of the sinusitis because of insufficient histopathological criteria, especially for cases 3–6, 8 and the present one. However, the association of the imaging findings, the positivity of both fungal culture and direct examination of sinus aspirate and/or sinus biopsy along with sinusitis symptoms was consistent with a sinus fungus ball for our case. Similarly, for cases 3–5,8 with multiple distant infection site, even if we could not conclude about the invasive character of the sinusitis, we suspected a secondary dissemination from a pulmonary infection site rather than from a sinus infection, based on clinical symptoms chronology.

The culture confirmed the diagnosis for all the cases. We highlight that at least one culture of sinus specimen (sinus biopsy, sinus aspirate) tested positive for *H. capsulatum* in most reported cases. This result contrasts with the low positivity rate of fungal culture (between 23 and 50%) observed from typical fungus balls, probably due to a poor viability of fungal elements in the fungus ball [8, 10]. This current gold standard diagnostic test presents a positivity rate of more than 75% cases in disseminated *Histoplasma capsulatum* infections [11]. However, the long delay for the fungi culture could easily allow the routine use of the qPCR test for rapid diagnosis. This has not been possible in this present case as the home-made qPCR is performed for research only. However, its sensitivity has been assessed in small cohorts of patients and different outcomes have been observed when compared with other diagnostic methods [11]. As an alternative to PCR, cross reactivity with *Aspergillus spp.* galactomannan antigens in sera have also been reported to be useful for the diagnosis of disseminated histoplasmosis in AIDS patients in endemic areas where *Histoplasma* EIA is not available [29, 30]. Unfortunately, the test was negative for our case and not used in the previous case reports. Recently, detection of specific *Histoplasma capsulatum* galactomannan antigens in urine specimens during immunoenzyme assay (EIA) has been shown to be a promising test, highly sensitive and rapid [31]. This report highlights the need of new biological tools to improve and accelerate diagnosis of histoplasmosis.

### Treatment and outcome

Antifungal treatment of sinusitis in the previously reported cases consisted in a long-term administration of Amphotericin B (cases 1, 6, 8), Itraconazole (cases 2, 3,4,7) and Posaconazole (case 5) leading to resolution of symptoms. No local surgery was required in contrast to typical sinusitis fungus balls that require endoscopic sinus surgery [8, 10]. No death was reported. Recommended antifungal agents for histoplasmosis treatment are Amphotericin B (including liposomal and lipid complex formulations) and Itraconazole [12]. Posaconazole and Voriconazole also demonstrated in vitro activity against *H. capsulatum* and most studies have reported success with these azoles [24, 32]. In present case, Voriconazole efficiency was difficult to evaluate as treatment was introduced too late with a curative dose for aspergillosis and the patient had too many concomitant life-threatening co-infections such as lymphoma and disseminated mycobacterium infection. This was the only *H. capsulatum* sinusitis case reporting patient death but association between *H. capsulatum* infection and death could not be demonstrated due to multiple co-morbidities.

In conclusion, histoplasmosis is a serious fatal risk for the AIDS-patients, requiring quick and reliable routine diagnostic methods. Moreover, there is a lack of specific recommendation for histoplasmosis prophylaxis in France, without any exception for French Guiana [33]. In opposition, in USA, a prophylaxis with Itraconazole (200 mg daily) is recommended in HIV infected patients with CD4 cell counts < 150 cells/mm3 in specific areas of endemicity [3]. A particular attention has to be given in AIDS-patients with sinusitis symptoms in *H. capsulatum* endemic areas such as in French Guiana. Despite the rarity of the clinical symptoms, sinusitis could represent an early expression of a *H. capsulatum* exposure. A sinus aspiration and/or a sinus biopsy should be performed rapidly for microbial analysis and essential as it can evolve into a disseminated infection when immunity decreases.

## Abbreviations

AIDS: Acquired Immunodeficiency Syndrome
Bal: Bronchoalveolar lavage
CHAR: Centre Hospitalier Andrée Rosemon
CT- Scan: Computed tomography scan
EIA: Enzyme immuno-assay
GG: Gomory Methanamine-Silver Grocott staining
HIV: Human immunodeficiency virus
MGG: May Grunwald Giemsa
PCR: Polymerase Chain reaction
